# Micro RNA210 expression in pregnancies with preeclampsia

**DOI:** 10.6026/97320630019319

**Published:** 2023-03-31

**Authors:** Shali Paremmal, Nidhi Sharma, Rama Devi, Keerti Gopi

**Affiliations:** 1 Department of Anatomy, Government Medical College Mahabubnagar, Telangana; 2 Department of obstetrics and gynaecology, Saveetha Medical College, Tamilnadu; 3 Department of Microbiology, Government Medical College Mahabubnagar, Telangana; 4 Department of Biochemistry, Government Medical College Mahabubnagar, Telangana India

**Keywords:** Preeclampsia, hypoxia, microRNA 210

## Abstract

Preeclampsia is one of the major causes of perinatal mortality and morbidity even in developed countries, the aetiology of which is not yet understood completely. In recent times, mi RNAs have gained prominence as regulators of the expressions of their target genes in health and pathological condition. mi RNA210, one of the important hypoxamirs, is reported to be a regulator of many cellular mechanisms including cell division, differentiation, apoptosis, cell cycle regulation, mitochondrial function, metabolism etc. Since hypoxia is the microenvironment that prevailed in preeclampsia it is worth full to see the expression pattern of mi RNA 210 as an attempt to unearth the preeclampsia pathogenesis. The placental tissue is collected from age-matched control and preeclamptic patients after strictly applying the inclusion and exclusion criteria. The present result shows 2.7 fold-up regulation of miRNA210 in preeclampsia in rt PCR study, the role of which need to be studied further to understand the pathogenesis of preeclampsia.

## Background:

The clinical presentation of Preeclampsia is characterized by hypertension and proteinuria developing after 20 weeks of pregnancy, which is a severe complication of human pregnancy with a worldwide incidence of 2-8% [1]. It is one of the main causes of maternal and perinatal morbidity and mortality, even in developed countries. Recent studies suggest that an excessive maternal systemic inflammatory response to pregnancy with activation of both the innate and adaptive arms of the immune system and an imbalance between circulating angiogenic factors and their antagonists plays a crucial role in the pathogenesis of the disease[2,3][25]. In addition, the development of preeclampsia is influenced by genetic and environmental risk factors suggesting its multi-factorial inheritance. The major pathologies identified in preeclampsia to date are endothelial dysfunction which is characterised by vascular hyper permeability, thrombophilia, and hypertension. These alterations evolve as a compensative mechanism for the decreased flow in the uterine arteries due to peripheral vasoconstriction. Endothelial dysfunction is responsible for the clinical symptoms observed in the mother, ie, impairment of the hepatic endothelium, contributing to the onset of HELLP (Hemolysis, Elevated Liver enzymes and Low Platelet count) syndrome, impairment of the cerebral endothelium inducing refractory neurological disorders, or even Eclampsia [4][26]. Further, depletion of vascular endothelial growth factor (VEGF) in the podocytes makes the endotheliosis a more efficient factor to block the slit diaphragms in the basement membrane, leading to decreased glomerular filtration and proteinuria VEGF and its receptors expressions are highly dis regulated in severe preeclampsia and HELLP syndrome [5]. Ultimately, endothelial dysfunction promotes micro angiopathic haemolytic anaemia, and vascular hyper permeability associated with low serum albumin-causing oedema, particularly in the lower limbs or lungs [24]. However, the pathophysiological mechanism of preeclampsia at cellular and molecular is very tough to define. In this context, the genetic and immunological theory has provided key insights to identify several susceptibility genes that probably interact in the haemostatic and cardiovascular systems, as well as in the inflammatory response in preeclamptic conditions [6]. However, more studies are required to unearth the key molecular mechanisms to understand the consequence of a series of cellular and molecular events at different signalling cascades associated with cellular functions like integrated stress pathways, hypoxia, angiogenesis, cell differentiation, cell cycle regulation, proliferation and growth, inflammation, DNA damage repair, mitochondrial metabolism and epithelial-to-mesenchymal transition (EMT), etc. In regulating the above cellular and molecular events, several key transcriptional and post-transcriptional factors play an important role which culminates in the expression/suppression of certain pathological conditions [7]. Among these microRNAs are recently identified non-coding genomic molecules that have the potential to regulate the expression of their target genes, sometimes in a tissue-specific and temporal manner. The abundance of their presence in the human placenta, where precise temporal regulation of gene expression plays an integral role in development, drew huge attention towards this area, of late [8]. Micro RNAs are a group of non-coding RNAs (ncRNA) that do not translate into protein. Discovered in 1993 as a small noncoding RNA, the micro RNA became a hot topic in the current research arena because of its regulatory effect on the expression of its target genes. Hundreds of mi RNA have been discovered through cloning and size-fractionated RNA techniques. Their regulatory role is diverse from cell proliferation, cell differentiation, cell death, fat metabolism, neuronal patterning and immunity. Micro RNAs play a unique part in placental development and in the progression of pregnancy in general. The aberrant expression and abnormal interactions of micro RNA are excessively studied in recent times due to their significant role in the mechanism of development of many clinical conditions including Preeclampsia. Several studies have suggested that mi RNAs are important regulators of Cytotrophoblast and Syncytiotrophoblast (STB) differentiation. Microarray analyses of mi RNA expression profiles in primary trophoblast before and after their differentiation into STB have revealed that multiple members of chromosome 19 microRNA cluster(C19MC) such as miR-515-5p, miR-518f, miR-519c-3p and miR-519e-5p were significantly down-regulated during CTB to STB differentiation [9][10]. However, the current understanding of functional regulation of placental-specific mi RNAs in development is in its infant stage.

Various studies show that a variety of cellular physiological functions like cell proliferation, differentiation, apoptosis, cell cycle regulation, mitochondrial function, metabolism, angiogenesis, neurogenesis, erythropoiesis and spermatogenesis are closely implicated with miR210. Recently, aberrant miR210 expressions profile in a pathological condition became the focus of multitudes of studies, which cover tumorogenesis, cancer, status epilepsy, cryptorchidism, and cardiovascular diseases (CVDs). MiR-210 is now earned the status of being a key hypoxia-response factor both in healthy and disease states, emphasising its name as the master 'hypoxamir'a hypoxia-inducible mi RNA [11]. miR210 up regulation in response to hypoxia has been successfully demonstrated in a variety of cell lines [11]. In addition to hypoxia, and inflammatory conditions, oxidative stress is also seen as a regulatory factor in the miR210 expression pattern, which can be seen as a hypoxia-independent contributor. Despite the wealth of studies which have suggested an association of certain mi RNAs with preeclampsia, no consensus has yet been established on which appears to be mostly contributed towards the actual pathway, through which mi RNAs regulate certain biological processes. Our hypothesis is that mi RNA 210 has differential expression profiles in preeclampsia, which could be a contributing factor towards the pathogenesis of this condition. Therapeutic regulation of miR-210 expression may therefore represent a potential treatment strategy.

## Materials and Methods:

## Subject selection:

The study is performed at Govt. Medical College Mahabubnagar, Telangana, India. The study was carried out after obtaining the Ethical Committee Clearance Certificate from the Institutional Ethical Committee and informed consent was taken from the patients and healthy controls. Placental tissues were taken from age-matched 45 preeclamptic cases and 15 normotensive pregnant women after delivery. The inclusion criteria for the selection of the samples comprise the following. De novo appearance of hypertension (SBP ≥140 mmHg or DBP ≥90 mmHg) and Proteinuria (≥0.3 g/24 h of urinary protein or ≥2 + reading on a dipstick) after the 20th week of gestation in normotensive women matched for maternal age and parity. Women were tested for normal liver function tests and are euthyroid with BMI <25 and with no evidence of any other endocrinopathy. Subjects with diabetes mellitus, ischemic heart disease, stroke, peripheral vascular disease, cardiac, renal, hepatic dysfunction, chronic hypertension, pre-existing seizure disorder, eclampsia, pre-gestational diabetes, placental abruption, gestational diabetes, thyroid disease, dyslipidaemia are excluded from the study.

## Sample collection:

Placental tissue samples were collected after the delivery and washed in phosphate-buffered saline (PBS) to remove blood contamination. 2-3gms of tissue from the foetal surface of the middle portion of the placenta is transferred to 1x phosphate buffer saline for molecular analysis and stored at -80 degrees till the experiment is carried out. The phenol/alcohol method was adopted for total mRNA isolation and isolated m RNA was stored at -20 degrees till further processes. cDNA synthesis of micro RNA is done using Universal Stem Loop Primer(USLP) and Universal 6 Reverse Primer(U6RP)[12].Quantification of miRNA210 was performed based on SYBR–Green assay using qRT-PCR. (Quant studio 5, Applied biosystem)

## Primers of miR-210 were designed as follows:

miR-210 - forward: 5-GTGCAGGGTCCGAGGT- 3, and

miR-210 - reverse: 5-CTGTGCGTGTGACAGCGGCTGA-3;

U6-forward: 5-CTCGCTTCGGCAGCACA-

U6- reverse: 5ACGCTTCACGAATTTGCGT-3.

Human microRNA-specific forward and reverse primers were used for the quantification of the samples. The following conditions were set for conducting qRT-PCR. Denaturation at 94 °deg;C for 2 min and 40 cycles of 94 °C for the 30s, 55-60 °C for 30s and 72 °C for 30s and melting curve of 10 min. The expression level of miR 210 is calculated using relative fold change in both controls and preeclampsia subjects in relation to the amount of U6Sn RNA present in the same sample. Each sample is performed in triplicate and the mean value is calculated.

## Results:

## Statistical analysis:

**Figure 1:**
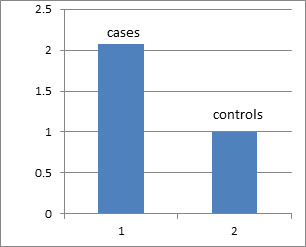
The analysis of the relative expression profile of the mi RNA210 showed a significantly increased expression level in pre-eclamptic placental tissue compared to the healthy controls. PE group has shown a fold change of 2.07 compared to the control group.

The change in expression levels of each mi RNA was analysed using the relative quantification method (RQ=2-ΔΔCT) as described by Livak method [13]. The graphs of all mi RNA levels have been represented as Ln < delta< Ct. One sample t-test was applied for column statistics to find significance between control and patient groups. Data were analysed by Microsoft Excel and Graph pad prism software. Data were summarized by Mean ± SD for continuous data. The comparison between the two groups was done by unpaired t-test for continuous normal data and Mann-Whitney U test for continuous non-normal data. All p-values less than 0.05 were considered statistically significant (Figure 1).

## Discussion:

The present study showed a significant (>0.05) level of expression in miR210 in pre-eclamptic placental tissue in rt-pcr analysis comparison with the age-matched normal placenta. Essentially the microenvironment for preeclampsia is hypoxia due to poor vascular remodelling of uterine arterioles. Recent studies have successfully established the correlation between HIF-1 α and microRNA expression in the hypoxic condition in colon and breast carcinoma cell lines[14].Hypoxic condition induces the upregulation of miRNA210 in human placental choriocarcinoma JAR cell lines [15]. Among the hypoxia-regulated micro RNAs, miR210 is widely studied for its involvement in pathological conditions. MiR-210 is the most studied hypoxamir, which is upregulated directly by HIF1A[16].Several studies have shown that hypoxia induces HIF1 transcription factor which in turn regulates downstream targets, namely soluble vascular endothelial growth factor receptor-1 (sFlt-1), transforming growth factor beta, and Endoglin and all these found to be involved in the clinical manifestation of PE. In vitro studies have shown that HIF-1α binds to the HRE region of the miR210 promoter region in the trophoblastic Jar cells and human umbilical vein endothelial cells (HUVECs). Studies have also provided broader evidence of a hypoxia-dependent induction of miR-210 by demonstrating upregulation of the miRNA in response to hypoxia in practically all primary cells and cell lines, which includes trophoblast-derived cell lines, primary trophoblasts, and ex vivo uterine arteries. miR-210 is involved in the regulation of hepatocellular carcinoma via HIF-1α and HIF-3α[17].Again, miR-210 is a target of HIF-1 and HIF-2 and is closely correlated with the prognosis of patients with renal cancer [18]. Collectively, these studies support that hypoxia, potentially through induction of HIF-1α, up-regulates miR-210 expression. It is an interesting observation that, in breast cancer, hypoxia induces miR 210 upregulation through HIF-1alpha/ von Hippel-Lindau (VHL) transcriptional system but not HIF-2alpha [19].The expression of HIF1a has been reported to be upregulated in preeclamptic placentas obtained by caesarean section [20].Apart from HIFs, the is also regulated by the hypoxia-responsive transcription factor, nuclear factor kappa-B subunit p50 (NFKB1), in primary human trophoblasts [21].In vitro studies report this transcription factor binds to the miR-210 region in primary trophoblasts and JAr cells, supporting the data showing increased NF-κBp50 and miR-210 levels seen in the placentas of the TLR3-induced PE mouse model. Immuno histo-chemical studies of PE show higher expression of NF-κBp50 and NF-κBp65 in placentas from pregnancies complicated with PE.

N(gamma)-nitro-L-arginine methyl ester(L-NAME)-induced preeclamptic rats also displayed enhanced NF-κB activation and lipopolysaccharide (LPS)-induced preeclamptic rats showed elevated levels of NF-κBp65. In agreement with the general trend toward upregulation of NF-κB transcriptional factors, results of Immuno histochemical studies conducted in placentas from pregnancies complicated with PE show higher expression of NF-κBp50 and NF-κBp65. Enhanced NF-κBp50 expression and activation in the placenta therefore probably plays a role in the pathology of PE and contributes to the upregulation of miR-210 in PE. Studies showed that subjects with elevated serum miR210 in the second trimester of pregnancy developed PE during the later stage of pregnancy [22]. Transfection studies of extra villus trophoblast cells, from 1ST trimester placental tissue, using miR210 mimics, by the same team, also showed an upregulation in miR210, concluded that even though the molecular mechanism by which the miR210 regulate the trophoblastic invasion is a complex one, there are multiple pathways including MAPK signalling depended on the manner through which miR210 interfered with trophoblastic invasion. A previous study by the same team had shown that Lipopolysaccharide (LPS) inhibited trophoblast invasion in MAPK dependent manner [23]. Further, in a murine macrophage study, LPS seen to activate miR210 expression.

## Conclusion:

In short, miR210 serves as a node for multiple stimuli, interactions of which regulate the trophoblast invasion. The increased expression of miR-210 in the placenta of women with preeclampsia suggests a possible functional role in the pathophysiological characteristics of this disease.
